# A case study evaluation of competitors undertaking an antarctic ultra-endurance event: nutrition, hydration and body composition variables

**DOI:** 10.1186/s13728-015-0022-0

**Published:** 2015-03-12

**Authors:** Scott Paulin, Justin Roberts, Michael Roberts, Ian Davis

**Affiliations:** School of Life and Medical Sciences, University of Hertfordshire, College Lane, Hatfield, Hertfordshire AL10 9AB UK; Department of Life Sciences, Anglia Ruskin University, Cambridge Campus, East Road, Cambridge, CB1 1PT UK; National Health Service, Rendcomb, Cirencester, Gloucestershire GL7 7EY UK

**Keywords:** Ultra-endurance, Antarctica, South Pole, Nutrition

## Abstract

**Background:**

The nutritional demands of ultra-endurance racing are well documented. However, the relationship between nutritional consumption and performance measures are less obvious for athletes competing in Polar conditions. Therefore, the aim of this study was to evaluate dietary intake, hydration status, body composition and performance times throughout an 800-km Antarctic race.

**Methods:**

The event organisers declared that 17 competitors would participate in the South Pole race. Of the 17 competitors, pre-race data were collected from 13 participants (12 males and 1 female (M ± SD): age: 40.1 ± 8.9 years; weight: 83.9 ± 10.3 kg; and body fat percentage: 21.9 ± 3.8%). Dietary recall, body composition and urinary osmolarity were assessed pre-race, midway checkpoint and end race. Data were compared on the basis of fast finishers (the Norwegian team (*n* = 3) who won in a record of 14 days) and slower finishers (the remaining teams (*n* = 10) reaching the South Pole between 22 and 28 days).

**Results:**

The percentage contribution of macronutrients to daily energy intake for all participants was as follows: carbohydrate (CHO) = 23.7% (221 ± 82 g.day^−1^), fat = 60.6% (251 ± 127 g.day^−1^) and protein = 15.7% (117 ± 52 g.day^−1^). Energy demands were closer met by faster finishers compared to slower finishers (5,332 ± 469 vs. 3,048 ± 1,140 kcal.day^−1^, *p* = 0.02). Average reduction in body mass throughout the race was 8.3 ± 5.5 kg, with an average loss of lean mass of 2.0 ± 4.1 kg. There was a significant negative correlation between changes in lean mass and protein intake (*p* = 0.03), and lean mass and energy intake (*p* = 0.03). End-race urinary osmolarity was significantly elevated for faster finishers compared to slower finishers and control volunteers (faster finishers: 933 ± 157 mOsmol.L^−1^; slower finishers: 543 ± 92 mOsmol.L^−1^; control: 515 ± 165 mOsmol.L^−1^, *p* = 0.04).

**Conclusions:**

Throughout the race, both groups were subjected to a negative change in energy balance which partly explained reduced body mass. Carbohydrate availability was limited inferring a greater reliance on fat and protein metabolism. Consequently, loss in fat-free mass was more prevalent with insufficient protein and caloric intake, which may relate to performance.

## Background

Understanding the role of nutrition and associated dietary demands within endurance sports has grown in popularity particularly in recent years [1-9]. The potential benefits of dietary and nutrient intake for elite and non-elite endurance athletes are well documented [3]. Adventure races are typically characterised as non-stop races or multistage races over multiple days. The duration and training volume (distance) of events can range between 17 h for an ironman triathlon to 28 days, as was the case for the Antarctic South Pole Challenge event. The unique physical demands associated with ultra-endurance racing (multi-day duration, different modes of physical activities, climatic conditions associated with the race location, terrain, navigation and survival skills and sleep deprivation) affect the nutrition demands for the participating athletes; however, details regarding nutritional requirements and how these might differ between events are still not fully understood.

A previous research investigated ultra-marathoners running over 160 km in the heat [2]. Of the 23 athletes assessed, 13 successfully completed the race. Finishers were found to ingest higher amounts of fluid (19.4 ± 8.1 L) and sodium (16.4 ± 9.5 g) than non-finishers [2]. Serum sodium concentration was lower in the mid-phase of the race in those individuals experiencing negative changes in mental status. Whilst caloric demands were more closely met in the finishers compared to the non-finishers, it is evident that further understanding of macronutrient demands during such events is warranted, especially when races are conducted over multiple days.

To date, there are limited studies that have documented the dietary requirements and food selections for ultra-endurance athletes racing in cold weather conditions [7,9,10]. In a recent review, the dietary practices for two men attempting an unsupported speed crossing expedition of Greenland were reported [9]. The 2-kg daily rations for the Greenland crossing provided 36.0 MJ.day^−1^ (8,600 kcal.day^−1^) of energy [9]. Interestingly, during the Michael Stroud/Ranulph Fiennes (MS/RF) crossing of Antarctica in the early 1990s, the two men carried rations which averaged 21.3 MJ.day^−1^ (~5,000 kcal.day^−1^) [10]. The discrepancies between daily energy consumption and the percentage contribution of macronutrients towards daily energy intake for both the Greenland (63% CHO, 26% fat and 11% protein) and MS/RF crossing of Antarctica (35% CHO, 57% fat and 8% protein) are likely to accommodate the differences in expedition duration and exercise intensity.

It is also evident that for shorter, higher intensity ultra-events such as Ironman triathlon and the Tour de France stages, carbohydrates appear to be the preferred energy source for athletes [11]. Interestingly, there is much inconsistency surrounding the quality and quantity of chosen foods during ultra-endurance events. Bananas, biscuits, energy gels/bars and sports drinks are popular food and fluid choices for shorter endurance and ultra-endurance events. However, as the duration of the race increases, these food and drink choices become less appealing or tolerable [4,8]. Consequently, athletes may under fuel which, in part, may explain why energy expenditure exceeds energy intake during adventure racing. Other factors that influence extreme negative energy balance during ultra-endurance events may relate to difficulty carrying or towing food and fluid supplies, general pack weight constraints, lack of storage points and gastrointestinal distress issues [4]. In contrast, regarding the choices of foods, there is an apparent shift towards foods rich in protein and fat during longer lasting ultra-endurance races. Naturally during multi-day events, exercise pacing tends to conform to submaximal levels of intensity, often below lactate threshold, in order to preserve limited glycogen stores and optimise fat utilisation and the Krebs cycle pathway for ATP resynthesis [11].

Partly due to the financial and political issues in gaining access to Antarctica, no study to our knowledge has conducted research on athletes in a competitive race across Antarctica. Therefore, this case study evaluation aimed to profile nutritional intake, hydration status, body composition and performance times throughout an 800-km race across Antarctica. It was hypothesised that energy, fat and protein intake would be greater with faster finishers due to increased energy demands as a result of faster pacing. Due to the intensive demands of the event, it was also hypothesised that hydration status would be more affected in faster finishers at the end of the race.

## Case presentation

### The race

Event organisers Extreme World Races Ltd (EWR) hosted a unique Centenary race to commemorate the 100th Anniversary of Captain Robert Falcon Scott’s ill-fated attempt to become the first explorer to reach the Geographical South Pole. Unfortunately, Captain Scott’s journey was in vain as Norwegian explorer Roald Amundsen and his crew arrived at the South Pole in December 1911, 4 weeks before Scott. On the 4th January 2012, 17 athletes contested an 800-km adventure race aiming to reach the South Pole finishing line located at the American South Pole Research Station.

Unlike most adventure races where there are a number of allocated transition points that the athletes must reach at the end of each stage, athletes in this race were completely self dependent, with the exception of the 400-km midway transition checkpoint. This meant the athletes were responsible for towing their own camping supplies (i.e., tents, cooking equipment, sleeping bags etc.), food and fluid supplies and dry clothing. Each athlete towed their own equipment and supplies in a large pulk or sledge that was attached to the athlete via a safety harness. At all times during racing hours, athletes wore insulated clothing suitable for Antarctic conditions. Food was limited to how much each athlete was able to tow. The midway checkpoint provided an excellent opportunity where athletes could resupply their pulks, refuel and seek medical attention from the race doctor if needed. Each team and individual within that team was responsible for their own food selections and energy intake targets throughout each racing day.

### Methods

The study was granted local approval by the University of Hertfordshire Life and Medical Sciences Ethics Committee. All participants provided written, informed consent and completed a health screen questionnaire prior to study inclusion. A medical history questionnaire was used to investigate past medical history and current health status prior to the event.

#### Participant information

Before departing to Antarctica, the event organisers declared that 17 individuals would be racing to the South Pole. Of the 17 competitors, body composition variables were collected from 13 participants (12 male and 1 female) as follows: (mean ± SD) age = 40.1 ± 8.9 years, weight = 83.9 ± 10.3 kg and body fat percentage = 21.9 ± 3.8%) (Table 1). A team of two competitors had to be evacuated during the acclimation stage of the event, whereas another team of two competitors did not meet the study requirements. Additionally, when the competitors where requested to provide recall diaries throughout the race, only ten competitors complied. Urinary specimens were successfully collected from all participants at the designated data collection points between the start and finish of the 800-km event. In addition, five crew members voluntarily gave urine specimens for control purposes. Competitors were divided into either faster (*n* = 3) or slower finishers (*n* = 10). The faster finishers completed the 800-km race in a record time of 14 days. All other teams reached the finishing line in close succession in terms of days between race days 22 and 28 (Table 2).

**Table 1:** **Mean competitor characteristics at the start line and finishing line (** ***n*** **= 13)**

**Variables**	**Start line**	**Finishing line**
Age (years)	40.1 ± 8.9	
Weight (kg)	83.9 ± 10.3	75.8 ± 7.4
Body fat (%)	21.9 ± 3.8	16.2 ± 5.0
Fat mass (kg)	18.7 ± 3.9	12.5 ± 3.9
Lean body mass (kg)	66.5 ± 9.3	64.5 ± 7.7
Waist circumference (cm)	90.8 ± 6.6	78.2 ± 10.2
Thigh circumference (cm)	58.2 ± 4.0	53.6 ± 2.5

**Table 2:** **Race performance and team data**

**Race positions**	**Team names**	**Finishing times (days)**
1	Framdrift	14
2	Team mercury	22
3	Mission possible	26
4	Centrepoint	28
D*	EBB	23
D*	Keep a child alive	28
DNF^#^	Sladenwoods	DNF

*Team disqualified due to a breaching of rules, but permitted to continue skiing. ^#^Team did not finish.

#### Urinary collection and analysis

Urinary specimens were collected, analysed and stored (below −20°C) in duplicate on race day morning, the midway checkpoint and South Pole finishing line. Each competitor was asked to provide a 40-mL urine sample on three separate occasions. The control group complied with a similar collection protocol; however, urinary samples were obtained after an overnight fast (before breakfast) and collected immediately after urination.

#### Urinary sodium analysis

Immediately after urination, urinary electrolyte status was assessed in duplicate using a portable ion meter (SG8 SevenGo Pro pH/ion meter, Mettler-Toledo Ltd., Leicester, UK) for sodium (Na^+^) concentrations in a pre-heated sheltered environment before freezing in outdoor conditions (<−20°C). The urine samples remained frozen and were stored in a large cooler box for later comparative analysis under laboratory conditions.

#### Urinary osmolarity analysis

Assessment of urinary osmolarity was conducted post-race under laboratory conditions using a freezing point depression osmometer (Micro-Digital Osmometer Type 6, Camlab Ltd., Cambridge, UK). Following manufacturer recommendations, it was deemed that the osmometer was not robust enough to cope with field conditions. It is worth highlighting that urine specimens were analysed for ketone presence using a dipstick test. However, the results were not valid due to technical issues and therefore were not included.

#### Body composition analysis

Body mass and composition data were collected at the corresponding time points in a pre-heated oven tent. Whole body mass was quantified using a set of standard body weight scales (Seca 875 flat weighing scales, Seca, Birmingham, UK). To avoid displacement due to the undulating surface of the inland plateau, the scales were placed on a flat sheet of plywood. A spirit level was used to measure the equilibrium of the surface where the body weight scales would be used for assessment. Skin folds were measured in millimetres using a set of precision anthropometrical callipers (Harpenden Skinfold Caliper, Baty International Ltd., West Sussex, UK). To avoid possible cold burns, the steel callipers were warmed in a pre-heated tent for 5 min prior to use. Skin fold measures were taken in triplicate (on the right hand side of each participants’ body, whilst standing in the anatomical position) from the bicep, triceps, subscapular and suprailiac regions in accordance with the American College of Sports Medicine (ACSM) guidelines for anthropometrical assessment. Body fat percentage, fat mass and fat-free mass were calculated using the Durnin/Womersley equation [12]. A standard measuring tape was used to determine body circumference data from the waist and thigh.

#### Dietary collection and analysis

Participants were requested to record dietary intake on a daily basis for the duration of the Antarctic event using a recall method, with detailed instructions provided during the study briefing. Each participant was provided with a small notepad and pencil before the race. At the end of each racing day, competitors were asked to recall the quantity (using household measures) and the different types of food consumed each day. Dietary analysis for caloric, macronutrient and micronutrient intake was performed using a suitable software package (Training Peaks dietary software, Peaksware LLC, CO, USA).

#### Environmental information

The Arctic Truck ‘Gabrielle’ was equipped with automatic weather station measuring wind speed (m.s^−1^), wind gust (m.s^−1^), humidity (%) and air temperature (°C). These parameters were recorded (every 10 min) throughout the duration of the 800-km race. Average environmental data throughout the event were the following: wind speed 6.6 m.s^−1^, wind gust 8.8 m.s^−1^, temperature −24.0°C and 59.3% humidity. Altitude between the start line and South Pole finishing line ranged between 2000 and 2,615 m a.s.l.

#### Race location and additional information

The coordinates of the 2012 South Pole Challenge start line were 83° 43.261’ S 021° 06.661’ E. There was a compulsory 24-h stopover at the midway stage of the event. This provided the athletes an opportunity to recover, refuel and seek medical attention if needed. Once leaving the midway checkpoint, the race continued until all teams reached the South Pole finishing line. Coordinates for the checkpoint were 87° 00.077’ S 010° 18.493’ E.

#### Statistical analyses

Statistical analyses were performed using SPSS Statistics for Windows version 20 software (IBM Corporation, New York, US). Data were assessed for normality using a Sharpiro-Wilk test. Accordingly, a non-parametric Mann-Whitney *U* test was employed to examine differences between groups. Relationships were assessed using Spearman’s rho test. An alpha level of *p* ≤ 0.05 was employed for statistical significance. Data are reported as means ± SD.

### Results

#### Macronutrient and energy intake during the race

During the Antarctic event, 10 of the 17 competitors complied dietary recall diaries every 24 h for the duration of the event. The percentage contribution of macronutrients to daily energy intake for the entire group was CHO = 23.7% (221 ± 82 g.day^−1^), fat = 60.6% (251 ± 127 g.day^−1^) and protein = 15.7% (117 ± 52 g.day^−1^). There was a significant difference reported for energy intake between groups (faster finishers: 5,332 ± 469 vs. slower finishers: 3,048 ± 1,140 kcal.day^−1^; *p* = 0.02) (Table 3). The percentage contribution of macronutrients to daily energy intake for faster finishers was CHO = 17.4%, fat = 66.5% and protein = 16.1%. For the slower finishers, the percentage contribution of macronutrients was CHO = 28.6%, fat = 56.1% and protein = 15.3%. Mean fat and protein intakes were significantly different between faster and slower finishers (fat: 394 ± 1 vs. 190 ± 99 g.day^−1^ (*p* = 0.02) and protein: 172 ± 5 vs. 93 ± 34 g.day^−1^ (*p* = 0.03)). Carbohydrate intake was marginally higher in the faster finishers group (230 ± 61 g.day^−1^) compared to the slower finishers (217 ± 94 g.day^−1^), but was not statistically different (*p* = 0.59).

**Table 3:** **Mean macronutrient and energy intake trends between faster (** ***n*** **= 3) and slower finishers (** ***n*** **= 10)**

**Daily macronutrient and energy intake**	**Fast finishers**	**Slow finishers**
CHO (g.day^−1^)	230.9 ± 61.1	217.7 ± 94.2
Fat (g.day^−1^)	394.3 ± 0.5*	190 ± 99.6
Protein (g.day^−1^)	172.1 ± 45.8*	93.5 ± 34.5
Energy (kcal.day^−1^)	5,332.5 ± 469.1*	3,048.9 ± 1,140.4

*Significant difference between groups, *p* < 0.05.

#### Relationships between changes in body composition, macronutrient intake and race performance times

No relationships were reported between changes in absolute body weight (*p* = 0.27), lean mass (*p* = 0.19) and race performance times. Additionally, there were no significant relationships between macronutrient intake and race performance times.

#### Macronutrient intake relative to bodyweight

Relative to total body mass, fat intake ranged between 0.6 and 4.8 g.kg^−1^.day^−1^; protein: 0.5 and 2.4 g.kg^−1^.day^−1^; and CHO 1.6 and 5.3 g.kg^−1^.day^−1^ (Table 4). There were no significant differences between faster and slower finishers for CHO (2.7 ± 0.7 vs. 2.7 ± 1.7 g.kg^−1^.day^−1^, *p* = 0.18) and protein (2.0 ± 0.6 vs. 1.1 ± 0.5 g.kg^−1^.day^−1^, *p* = 0.18) when intakes were adjusted for body weight. Significance was reported between faster and slower finishers when fat intake was adjusted for body weight (4.7 ± 0.1 vs. 2.3 ± 1.1 g.kg^−1^.day^−1^, *p* = 0.02).

**Table 4:** **Mean macronutrient intakes relative to body weight for faster and slower finishers during the race (** ***n*** **= 10)**

**Daily macronutrient intake relative to body weight**	**Faster finishers**	**Slower finishers**
CHO (g.kg^−1^.day^−1^)	2.7 ± 0.7	2.7 ± 1.7
Fats (g.kg^−1^.day^−1^)	4.7 ± 0.1*	2.3 ± 1.1
Protein (g.kg^−1^.day^−1^)	2.0 ± 0.6	1.1 ± 0.5

*Significant difference between groups, *p* < 0.05.

#### Assessment of vitamin, mineral and antioxidant nutrients between fast and slow finishers

Vitamin A (retinol) intake was significantly lower for the faster finishers compared to the slower finishers (150.5 ± 0.1 vs. 293.2 ± 101.7 μg.day^−1^, *p* = 0.02). Vitamin E (16.3 ± 6.9 vs. 1.4 ± 0.4 mg.day^−1^, *p* = 0.017) and selenium (3,714.2 ± 1,881.9 vs. 17.6 ± 5.3 μg.day^−1^, *p* = 0.02) were significantly higher within the faster finishers group compared to slower finishers. There were no reported significant differences between faster and slower finishers for vitamin C (2.1 ± 1.2 vs. 1.4 ± 0.4 mg.day^−1^) and zinc (10.7 ± 4.4 vs. 4.6 ± 1.3 mg.day^−1^, *p* > 0.05) (Table 5).

**Table 5:** **Mean antioxidant intake values during the race and RDA values for males (M) and females (F) (** ***n*** **= 10)**

**Antioxidant intake per day**	**RDA** [11]	**Fast finishers**	**Slow finishers**
Vitamin A retinol (μg.day^−1^)	0.9 mg (M), 0.7 mg (F)	150.5 ± 0.1*	293.2 ± 101.7
Vitamin C (mg.day^−1^)	90 mg (M), 75 mg (F)	2.1 ± 1.2	2.5 ± 2.9
Vitamin E (mg.day^−1^)	15 mg (M, F)	16.3 ± 6.9*	1.4 ± 0.4
Zinc (mg.day^−1^)	11 mg (M), 8 mg (F)	10.7 ± 4.4	4.6 ± 1.3
Selenium (μg.day^−1^)	55 mg (M, F)	3,714.2 ± 1,881.9*	17.6 ± 5.3

*Significant difference between groups, *p* < 0.05.

Trends of B vitamins were higher for the slower finishers compared to the faster finishers. Folate intake was significantly higher for the faster finishers compared to the slower finishers (72.4 ± 28.8 vs. 30.5 ± 7.9 μg.day^−1^, *p* = 0.02). There were no reported significant differences between the faster and slower finishers for thiamin (1.3 ± 0.6 vs. 1.4 ± 0.4 mg.day^−1^), riboflavin (0.7 ± 0.2 vs. 0.6 ± 0.2 mg.day^−1^), niacin (2.0 ± 0.8 vs. 4.7 ± 2.7 mg.day^−1^), pantothenic acid (0.8 ± 0.2 vs. 1.3 ± 0.3 mg.day^−1^) and vitamin B_12_ (0.3 ± 0.1 vs. 0.8 ± 0.3 μg.day^−1^) (Table 6).

**Table 6:** **Mean intakes of B vitamins and folate during the Antarctic event and recommended daily allowance values (RDA) for males and females (** ***n*** **= 10)**

**Vitamin intake per day**	**RDA** [11]	**Fast finishers**	**Slow finishers**
Thiamin (mg.day^−1^)	1.2 mg (M), 1.1 mg (F)	1.3 ± 0.6	1.4 ± 0.4
Riboflavin (mg.day^−1^)	1.3 mg (M), 1.1 mg (F)	0.7 ± 0.2	0.6 ± 0.2
Niacin (mg.day^−1^)	16 mg (M), 14 mg (F)	2.0 ± 0.8	4.7 ± 2.7
Pantothenic acid (mg.day^−1^)	5 mg (M, F)	0.8 ± 0.2	1.3 ± 0.3
Vitamin B6 (mg.day^−1^)	1.3 mg (M, F)	0.5 ± 0.1	0.4 ± 0.1
Folate (μg.day^−1^)	400 μg (M, F)	72.4 ± 28.8*	30.5 ± 7.9
Vitamin B_12_ (μg.day^−1^)	2.4 μg (M, F)	0.3 ± 0.1	0.8 ± 0.3

*Significant difference between groups, *p* < 0.05.

There were no reported significant differences between faster and slower finishers for calcium (848.7 ± 137.6 vs. 872.4 ± 260.5 mg.day^−1^), iron (8.1 ± 3.6 vs. 5.8 ± 2.0 mg.day^−1^) or manganese (4.8 ± 1.6 vs. 3.3 ± 1.5 mg.day^−1^). Sodium intake was, however, significantly higher for the slower finishers compared to the faster finishers (354.2 ± 38.4 vs. 3,769.4 ± 1,764.1 mg.day^−1^, *p* = 0.02). Intakes of phosphorus (1,859.3 ± 781.4 vs. 717.9 ± 200.2 mg.day^−1^, *p* = 0.02), potassium (1,747.2 ± 845.4 vs. 531.5 ± 152.3 mg.day^−1^, *p* = 0.02), magnesium (852.6 ± 407.7 vs. 215.2 ± 73.9 mg.day^−1^, *p* = 0.02) and copper (3.8 ± 1.8 vs. 0.9 ± 0.3 mg.day^−1^, *p* = 0.02) were significantly higher for faster finishers compared to slower finishers (Table 7).

**Table 7:** **The average intakes of minerals during the Antarctic event and the recommended daily allowance values (RDA) for males and females (** ***n*** **= 10)**

**Mineral intake per day**	**RDA [** 11 **]**	**Fast finishers**	**Slow finishers**
Calcium (mg.day^−1^)	1,000 mg (M, F)	848.7 ± 137.6	872.4 ± 260.5
Phosphorus (mg.day^−1^)	700 mg (M, F)	1,859.3 ± 781.4*	717.9 ± 200.2
Iron (mg.day^−1^)	8 mg (M), 18 mg (F)	8.1 ± 3.6	5.8 ± 2.0
Sodium (mg.day^−1^)	1,500 mg (M, F)	343.2 ± 38.4	3,769.4 ± 1,764.1*
Potassium (mg.day^−1^)	4,700 mg (M, F)	1,747.2 ± 845.4*	531.5 ± 152.3
Magnesium (mg.day^−1^)	420 mg (M), 320 mg (F)	852.6 ± 407.7*	215.2 ± 73.9
Copper (mg.day^−1^)	0.9 mg (M, F)	3.8 ± 1.8*	0.9 ± 0.3
Manganese (mg.day^−1^)	2.3 mg (M), 1.8 mg (F)	3.3 ± 1.5	4.8 ± 1.6

*Significant difference between groups, *p* < 0.05.

#### Assessment of body composition and macronutrient and energy intake

The average weight lost throughout the race was 8.1 ± 4.8 kg (range 1.7–15.4 kg) (*n* = 13). No significant differences were observed between faster and slower finishers for changes in absolute body weight (3.5 ± 1.6 vs. 10.5 ± 5.8 kg), lean mass (−1.9 ± 2.0 vs. 3.7 ± 4.0 kg), body fat percentage (5.8 ± 2.1 vs. 5.8 ± 2.0%), amount of body fat (5.6 ± 1.1 vs. 6.5 ± 1.9 kg), waist circumference change (5.7 ± 4.1 vs. 9.2 ± 5.3 cm) or thigh circumference change (3.9 ± 2.3 vs. 5.8 ± 3.3 cm).

Changes in absolute weight within groups ranged between 1.7 and 4.8 kg for faster finishers and 0.4 and 15.4 kg for slower finishers. Furthermore, changes within groups ranged between −0.4 and −4.1 kg (faster finishers) vs. −3.2 and −7.6 kg (slower finishers) for lean mass and 4.4 and 6.6 kg (faster finishers) vs. 3.2 and 8.2 kg (slower finishers) for body fat lost (kg).

**Figure 1:**
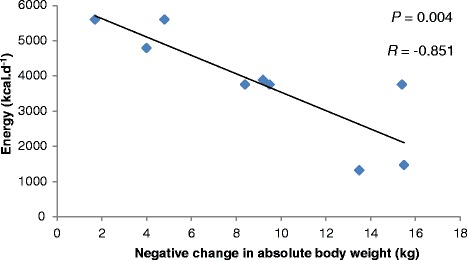
**The relationship between mean energy intake and changes in absolute body weight for male athletes (** ***n*** **= 9).**

**Figure 2:**
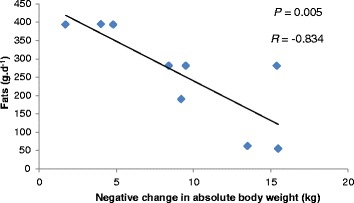
**The relationship between mean fat intake and changes in absolute weight for male athletes (** ***n*** **= 9).**

No relationships were reported between change in absolute body weight, macronutrient (CHO, *p* = 0.285; fat, *p* = 0.12; protein, *p* = 0.14) and energy intake (*p* = 0.11) when the total group data was statistically analysed. Interestingly, when the data was adjusted for male athletes only (*n* = 9), there were significant relationships between change in absolute body weight and fat, protein and energy intakes, respectively (Figures 1 and 2).

The average loss of lean mass throughout the race was 2.0 ± 4.1 kg (*n* = 13). There was a significant inverse correlation between change in lean mass, protein (*p* = 0.03) and energy intake *p* = 0.03). No relationship was reported between change in lean mass, CHO and fat intake.

#### Trends in urinary osmolarity during the race

**Figure 3:**
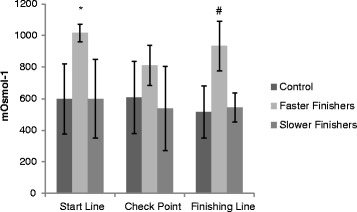
**Mean between group differences in urine osmolarity values at the start line, checkpoint and finishing line.**

Urine assessments were compared between three groups: control (*n* = 5), faster (*n* = 3) and slower (*n* = 12) finishers. Osmolarity measures between groups at the start line were significantly different (control: 599 ± 222 mOsmol.L^−1^; faster finishers: 1,017 ± 56 mOsmol.L^−1^; and slower finishers: 600 ± 249 mOsmol.L^−1^) (Figure 3). Post hoc assessment identified a significant difference between faster and slower finishers (*p* < 0.01) and faster finishers and controls (*p* = 0.04). No significance was reported between slow finishers and controls at the start of the race. No significant differences were reported between groups at the midway checkpoint (control: 608 ± 229 mOsmol.L^−1^; faster finishers: 812 ± 127 mOsmol.L^−1^; and slower finishers: 538 ± 266 mOsmol.L^−1^).

At the South Pole finishing line, urinary osmolarity was significantly different between groups (controls: 515 ± 166 mOsmol.L^−1^; faster finishers: 934 ± 158 mOsmol.L^−1^; and slower finishers: 544 ± 93 mOsmol.L^−1^, *p* = 0.04). Post hoc assessment identified a significant difference between faster and slower finishers (*p* = 0.05) only. Within each group, no significant differences were reported for urinary osmolarity over time.

#### Trends in urinary sodium concentrations during the race

Urinary sodium concentrations within the control group increased above baseline values at the checkpoint, before decreasing below baseline at the finishing line (3,008 ± 984 vs. 3,656 ± 1,277 vs. 2,424 ± 760 ppm). There were no significant differences reported regarding changes in urinary sodium within the control group throughout the event. For the faster finishers, urinary sodium increased at the midway checkpoint (4,025 ± 754 vs. 5,329 ± 1,616 ppm, *p* > 0.05), before significantly declining at the finishing line (3,130 ± 1,485 ppm) in comparison to the midway checkpoint (*p* < 0.01). There were no significant differences recorded between groups at the start line (*p* = 0.38), midway checkpoint (*p* = 0.35) and finishing line (*p* = 0.19).

### Discussion

The aim of this case study evaluation was to profile athlete dietary trends and investigate the relationship between dietary intake, hydration status, body composition and performance times throughout an 800-km race across Antarctica. The main findings from the study were as follows: 1) a significant negative correlation was observed between changes in absolute body mass, fat, protein and energy intake and 2) there was a clear significant relationship displayed between changes in lean mass, protein and energy intakes. The data suggest that total body mass and lean mass are better maintained when fat, protein and energy demands are closer met.

In most studies on ultra-endurance activities, a common finding is a high body mass loss occurring throughout the event. Body mass can be lost in a number of ways such as a loss of fluid, glycogen stores, lean muscle mass, stored body fat and bone mineral density. Interestingly, the percentage contribution of protein to daily energy intake was similar for both faster and slower finishers (CHO: 17.4 vs. 28.6%; fat: 66.5 vs. 56.1%; protein: 16.1 vs. 15.3%). In contrast, during the MS/RF 1993 expedition, the percentage contribution of macronutrients to daily energy consumption (57% fat, 8% protein and 35% carbohydrates) was not too dissimilar to that from the current study particularly when compared to the slower finishers [10]. However it must be noted that the daily amount of energy consumed by MS/RF was considerably greater compared to the slower finishers associated with the present study. It remains clear that for athletes competing in an adventure race, the large degree of body mass lost is typically a combined result of limited food availability, increased energy expenditure, sleep deprivation, dehydration and unavoidably low atmospheric oxygen pressure [1-18]. It is important to address that the percentage contribution of macronutrients to daily energy intake data is of little importance without the knowledge of the daily energy intake. Energy demands were closer met in faster finishers compared with slower finishers, a finding which might account for the greater weight loss in the slower finishers. It is worth mentioning that the slower finishers were exposed to the race conditionings for a longer time period therefore possibly contributing to the higher degree of body mass lost (slower finishers: 9.5 ± 4.6 vs faster finishers: 3.5 ± 1.6 kg).

In contrast, changes in body fat between groups were not too dissimilar between finishers. Therefore, the discrepancies in weight lost throughout the South Pole race between groups are likely associated with the differences in fat-free mass (FFM). Muscle degradation is a well-documented occurrence within endurance exercise [19]. The rate of muscle breakdown is accelerated when muscle protein oxidation exceeds synthesis, which usually occurs in proportion to intensity and duration of the sporting activity [10,19,20]. In addition, a low energy status and low intake of dietary protein particularly branched-chain amino acids (BCAA) contribute to the decline in muscle protein synthesis [14,20]. Regarding the regulation of protein synthesis from a cellular perspective, the mammalian target of the rapamycin pathway is a central point of control for muscle protein synthesis [19]. Sea-level studies indicate that acute caloric restriction (less than 3 weeks) activates AMP-activated protein kinase (AMPKα), an inhibitor of the rapamycin pathway [21]. As a consequence of low energy intake, protein availably too is limited. This suggests the importance for ingesting higher amounts of protein during intensive prolonged events [14]. Nevertheless, the dietary protein requirements for men undertaking competitive endurance exercise of this nature are still unclear. Nutritional intakes of 24 adventure racing athletes were profiled [4]. Protein intakes were high for both male and female athletes, respectively (1.9 ± 0.5 g.kg in men and 2.0 ± 0.4 g.kg in women). A previous research concluded that protein requirement in very active individuals may be as high as 160% of the RDA [19]. Whatever the case, protein intake relative to body weight within the current case study was 2.0 ± 0.6 g.kg for faster finisher and 1.1 ± 0.5 g.kg for slower finishers. Despite any significant value, there was a clear positive relationship reported between changes in lean mass and race completion times within the current study.

In the absence of sufficient carbohydrate availability during a prolonged endurance race, energy transfer is compromised [22]. In addition, there is an increased risk of becoming hypoglycaemic [5]. To maintain homeostatic functioning regarding the management of blood glucose levels, the body will rely on the process of gluconeogenesis, an alternative means for making new glucose molecules from a non-carbohydrate source. It is highly likely that amino acids derived from stored skeletal muscle were used to develop glucose molecules which therefore explain the discrepancies between changes in fat-free mass between faster and slower finishers.

Carbohydrate recommendations for endurance athletes vary throughout the literature [23-25]. In an Ironman triathlon study which investigated energy balance in male and female triathletes, carbohydrate intake relative to body mass ranged between 1.2 and 1.6 g.kg^−1^.h^−1^ [23]. Similar intakes have been report in shorter endurance events [24]. Remarkably carbohydrate intake relative to body mass between both groups of finishers in the current study was 2.7 g.kg^−1^.day^−1^. A rationale for the low carbohydrate intake reported is likely a result of limited access to food and possibly the impracticalities of stopping to eat at regular intervals throughout each race day. The South Pole athletes were advised before the race to snack throughout the event. The selections of foods for the ‘snack bags or day bags’ were of poor nutritional quality and consisted mainly of sweets, chocolate, nuts and biltong (Table 8). Feedback from the athletes following the event suggested that the food selections from the day bags became very monotonous and unsatisfying which resulted in less food being eaten across the group.

**Table 8:** **List of foods and beverages issued by the event organisers**

**Lists of foods**	**Lists of beverages**
Macadamias	Cadbury’s hot chocolate
Cadbury’s fruit and nut chocolate	Coffee
Cheddar cheese	Tea
Jelly Babies	Milk
Biltong	
Muesli	
Trek-and-eat dry meal	
Noodles	
Cup-a-soup	

Exercise intensity and duration will also influence the percentage contribution of macronutrients for energy transfer [23]. During ultra-endurance events such as the Tour de France and Ironman triathlons, the intensity of exercise is much greater compared to the intensity of the South Pole race. This increase in intensity will therefore place greater reliance on carbohydrate availability and the glycolysis pathway. Typically, a stage at the Tour de France on average lasts around 3–4 h whereby the cyclists can cover 180+ km per stage. In contrast, whilst the intensity endured by the South Pole athletes was much less, a typical race day could last anywhere between 10 and 15 h per day. The faster finishers were skiing at a rate of 3–4+ km.h^−1^ for at least 15 h per day.

As energy and carbohydrate availability was insufficient and the level of intensity was relatively low, it is plausible that there was a greater contribution of fat metabolism during the Polar event. Urinary specimens were tested for ketone production using a dip stick method; however, the results were not valid due to technical issues and therefore not included in the study. It was however evident that faster finishers were in favour of a diet rich in fat. Therefore, the faster finishers could afford to carry less food supplies whilst increasing the power to weight ratio of the pulk. This also highlights an important point that the heavier the pulk, the greater the energy expenditure will be.

A common reason as to why participants undertake adventure racing is the appeal of competing in a challenging unique environment. The South Pole competitors were exposed not only to cold weather conditions but also to an increase in altitude. Hypoxia is perhaps the largest contributor to muscle wasting at altitude [14,15]. Evidence suggests that hypoxic exposure may impair muscle protein synthesis through the downregulation of the rapamycin pathway by the hypoxia-induced REDD1 gene [13]. Of particular importance is that this downregulation is independent of low energy status. With this understanding, future recommendations for athletes competing in ultra-endurance events at altitude, who wish to maintain body mass, should aim to better meet caloric demands, ensuring protein intake is high and rich in BCAA availability.

It is worth highlighting that the team which finished in second position recorded the highest values for losses in total body mass and lean mass. This suggests that a loss in functional weight does not necessarily impair performance times. Other factors such as navigation, camp routine, race strategy, fitness and diet will undoubtedly influence performance times during an ultra-endurance race in Antarctica.

The micronutrient values reported in the current study were recorded from food sources only. It is worth highlighting that all participating teams did report using a multivitamin supplement at some point throughout the event. Therefore, it is likely that any ingested micronutrient values were much higher and closer to the RDA values. The current study evaluated a range of antioxidant nutrients, vitamins and minerals. Notably, the majority of the micronutrient intakes recorded was below RDA values for both faster and slower finishers. The lower recorded values can be explained by the selection of foods provided by the event organisers that were included in the athletes’ daily ration bags (Table 6). Additionally, because all the athletes were in energy deficit, it is unsurprising that micronutrient values were so low.

The implications of insufficient long-term micronutrient intakes may lead to the development of clinical and subclinical issues. Micronutrients have an important role in energy metabolism and biochemical functions; however, the relationship between the pre-race intake of micronutrients and race performance times are still unclear. The relationship of pre-race intake of vitamins and minerals in the form of supplementation before a multistage ultra-endurance run and their effect on performance has been investigated [1]. In the 4 weeks before the run, 9 runners (45% of the total) ingested vitamin supplements and 12 athletes (60%) mineral supplements. Athletes with an intake of vitamins (152.8 ± 14.1 vs. 160.6 ± 14.6 h) and minerals (151.6 ± 14.5 vs. 165.3 ± 10.8 h) finished the race no faster than athletes without a supplemental intake of vitamins and minerals [1].

In the current study, RDA guidelines were closer met for vitamin E, zinc, phosphorous, magnesium and manganese intakes within the faster finishers group. In contrast, slower finishers better met the RDA guidelines for thiamin, calcium and sodium. The relationship between RDA and micronutrient values directly relates to the specific foods used by each athlete. For example, selenium intake was significantly higher for the faster finishers compared to slower finishers (3,714.2 ± 1,881.9 vs. 17.6 ± 5.3 μg.day^−1^). Much of the faster finisher’s diet consisted of foods rich in fat and protein such as nuts and cheese. The volume of foods consumed and the choice of foods likely explains why selenium intake was considerably higher for faster than slower finishers. Selenium deficiency has been linked to an increased risk of various degenerative diseases such as heart disease and cancer [26]. In recent years, selenium’s role as an antioxidant nutrient has attracted increasing interest [26]. It is widely accepted that the need for antioxidant nutrients increases during exercise when free-radical production is high [1].

Athlete hydration status was assessed during the race only by the measurement of urine osmolarity, as the recording of fluid intake and urine volume was impracticable. It was hypothesised that there would be a significant difference in urine osmolarity between groups. Faster finishers were reported to have significantly greater urine osmolarity than slower finishers at the South Pole. It has been suggested that when relationships between hydration status and performance (finishing times) are assessed, an increase in athlete dehydration (in hot conditions) often does not degrade performance times alone during shorter endurance events [16]. That was in agreement with our current findings as a significant negative correlation was observed between race performance times and urine osmolarity results. In contrast, fluid consumption by athletes competing at higher intensities in a 160-km race was greater than our own findings [2]. It is worth repeating that fluid intake was not monitored in the current study. Instead, the faster finishers did report ingesting ~7 L of fluid per day.

The faster finishers were in a greater state of dehydration during the South Pole race. The most logical explanation for this occurrence is that the faster finishers spent more time skiing per day (~15 h.day^−1^) whilst ingesting less than 7 L of fluid per day. Fluid storage was an issue in Antarctica. A common problem experienced by many of the athletes was that without adequate insulation, drinking fluids would freeze. In addition, stopping to melt snow was time-consuming; therefore, given the competitive nature of the race, many of the faster athletes decided to melt more snow in the evening before bed. Furthermore, urinary sodium significantly increased in the faster finishers group between the start of the race and checkpoint. Interestingly, urinary sodium concentrations in the current case study ranged between 27.9 and 337.8 mmol.L^−1^ (642 and 7,767 ppm). An earlier study on Ironman triathletes in temperate conditions reported an inverse relationship between serum sodium concentrations and percentage change in body weight [5], indicating that dehydration is associated with hypernatremia. Logically, hyponatremia is associated with an increase in body weight as a result of increased fluid intake [5].

Urinary sodium excretion data obtained within the current investigation displayed a similar trend to athletes in another study when exposed to an increase in altitude [27]. Common to both studies’ urinary sodium excretion increased above baseline at the midway point before decreasing below baseline value at the finishing line. It is claimed that there is a delayed response to altitude ≤2,500 m where the hypoxic ventilatory response causes blood pH to rise which results in a metabolic compensation in the form of increased urinary secretion of bicarbonate, which might therefore explain an increase in associated urinary sodium output [27].

The South Pole athletes reported urinating more than normal as the race progressed. This contradicts the antidiuretic effect reported in other studies. The heightened range in urinary sodium output observed in the present study may be a homeostatic reaction to an increase in respiratory alkalosis. Although urinary bicarbonate was not measured, the latter explanation likely explains the increase in urinary sodium as bicarbonate is typically paired with a positively charged sodium ion to form sodium bicarbonate.

#### Limitations of the present study

Quality of control proved to be a challenge throughout the multi-day event. Unlike the Race Across America and the Southern Traverse Adventure Race in New Zealand where athletes are accompanied by a support vehicle or, alternatively, had a team of researchers present at each transition, this was not the case for the South Pole Challenge [6,18]. Transport was limited by the availability of fuel for the ‘Arctic Trucks’. Furthermore, fuel supplies were precisely calculated to transport the support crew to the next fuel depot, which at times was more than 1,200 km away. Therefore, as the race progressed and teams became separated, it was impractical to travel back and forth to collect data other than for emergencies. Also, the support team constantly changed throughout the event with staff members moving between different camps, making it difficult to collect data and ensure reliability. Dietary control data was considered, but any data gathered proved invalid as the data was very sporadic. In addition, we did manage to obtain five urinary specimens from the same five support crew members. It became apparent that control data was easier to obtain when the requirements were less for the participants involved.

We would also like to highlight that the study lacked external validity due to the small sample size. Nevertheless, the data in the current case study provide a useful insight regarding the role of nutrition and the associated dietary demands involved with an adventure race in Antarctica. Most notably, it was difficult to attain athlete hydration status simply based on urinary data alone. Future research should therefore consider additional blood and saliva samples for a more comprehensive insight into athlete hydration status. Data relating to fluid intake were also extremely difficult to collect during the Antarctic adventure race. Due to the low temperatures, any stored fluid typically froze without adequate insulation.

The data collected were not corrected for circadian rhythm. During the summer months (December–February), Antarctica is subjected to 24-h daylight. Several teams decided to ski during night time hours as the snow was more compact thus making it easier to ski. When a team eventually arrived at the midway transition, teams were suffering from severe sleep deprivation, hunger and illness/injury, thus refusing to comply with the research requirements until the following day. Anthropometric data and urine samples were collected upon awakening and before breakfast whilst the athletes were in a fasted state, thus eliminating the influence of diet on the latter variables.

It is noteworthy that there is a lack of studies on adventure racing more so in Antarctic conditions. Not only do such events present a physical challenge but there are other considerations such as sleep deprivation, psychological stress, and challenging environmental conditions and terrain which can all effect nutritional requirements. Energy expenditure was not monitored, therefore making it difficult to calculate an exact energy deficit. In addition, to better profile dietary and body composition data, frequent daily access to competitors would be required. However, given that this was impractical, only three data collection opportunities existed.

## Conclusions

Extreme long-distance endurance racing can result in significant negative energy balance. In addition to the low energy status observed in the study, macronutrient availability was limited. The macronutrient contribution to energy transfer and muscle protein resynthesis would therefore have been compromised leading to losses in body mass and fat-free mass. A specific dietary trend was witnessed whereby faster finishers ingested food sources richer in fat and protein with less reliance on carbohydrates. These findings suggest that a diet rich in fat and protein is better suited to athletes competing over long distances at lower intensities, in order to better maintain fat-free mass.

Food palatability presented an issue throughout the South Pole race and is likely responsible for the lack of food consumed by the athletes. Evidently, the food provided for the race severely lacked nutritional value and variety. For future events, it should be recommended that athletes prepare a diet rich in fat (~4.7 g.kg^−1^.day^−1^) and protein (~2.0 g.kg^−1^.day^−1^) with a particular emphasis on a variety of sweet and savoury food selections. Future athletes may also want to consider selecting foods with greater nutritional value. There is however no evidence to suggest that calories obtained from foods of poorer overall nutrient value are any less beneficial than those derived from foods of higher nutrient quality.

## Consent

Written informed consent was obtained from the participants for publication of this Case Report.

## Abbreviations

RDA: Recommended daily allowance
M: Male
F: Female
EWR: Extreme World Races
CHO: Carbohydrates
MS: Dr Michael Stroud
RF: Sir Ranulph Fiennes
TP: Training Peaks, Peaksware LLC dietary software
DNA: Deoxyribonucleic acid
